# Prevalence and predictors of self-reported alcohol abuse and its association with other mental health conditions in the residents of Fort McMurray after multiple traumas

**DOI:** 10.1192/j.eurpsy.2023.817

**Published:** 2023-07-19

**Authors:** G. Obuobi-Donkor, R. Shalaby, B. Agyapong, E. Eboreime, V. Agyapong

**Affiliations:** 1 Psychiatry, Dalhousie University, Halifax; 2 Psychiatry, University of Alberta, Edmonton, Canada

## Abstract

**Introduction:**

People consume and abuse alcohol for varied reasons. Problematic alcohol use is associated with mental and physical health risks, while people exposed to multiple traumas may be more vulnerable to abusing alcohol.

**Objectives:**

To evaluate the prevalence and predictors of self-reported alcohol abuse among residents of Fort McMurray and explore the correlates of self-reported alcohol abuse with some mental health conditions.

**Methods:**

A cross-sectional study adopted an online questionnaire. Sociodemographic data, trauma exposure, and clinical characteristics were collected to identify the predictors of self-reported alcohol abuse. Data were analyzed using SPSS version 25 using cross-tabulations and logistic regression analysis.

**Results:**

Two hundred and forty-nine individuals received the survey link, of which 186 completed the survey, with a response rate of 74.7%. Most participants were females exposed to COVID-19 and either wildfire or flooding traumas. The prevalence of self-reported alcohol was 27.4%. Participants who desired mental health counselling were likely to self-report alcohol abuse (OR=3.017; 95% CI: 1.349-6.750). There was a significant association between self-reported alcohol abuse and self-rated moderate to high depression (X ^2^ = 4.783; p = 0.033) and anxiety symptoms (X ^2^ = 4.102; p = 0.047), and suicidal ideations or thoughts of self-harm (X ^2^ = 13.536; p = 0.001).

**Conclusions:**

Self-reported alcohol abuse is correlated with suicidal ideations, the desire to receive mental health counselling, and anxiety and depression symptoms. Therefore, initiatives to minimize mental health disorders are crucial to reducing alcohol abuse and promoting health among vulnerable populations.

**Disclosure of Interest:**

None Declared

